# Add-on Protective Effect of Pentoxifylline in Advanced Chronic Kidney Disease Treated with Renin-Angiotensin-Aldosterone System Blockade - A Nationwide Database Analysis

**DOI:** 10.1038/srep17150

**Published:** 2015-11-27

**Authors:** Ko-Lin Kuo, Szu-Chun Hung, Jia-Sin Liu, Yu-Kang Chang, Chih-Cheng Hsu, Der-Cherng Tarng

**Affiliations:** 1 Division of Nephrology, Taipei Tzu Chi Hospital, Buddhist Tzu Chi Medical Foundation, Taiwan; 2 School of Medicine, Tzu Chi University, Hualien, Taiwan; 3 Institute of Population Health Sciences, National Health Research Institutes, Taiwan; 4 Department of Health Services Administration, China Medical University, Taichung, Taiwan; 5 Institute of Clinical Medicine, National Yang-Ming University, Taipei, Taiwan; 6 Department and Institute of Physiology, National Yang-Ming University, Taipei, Taiwan; 7 Division of Nephrology, Department of Medicine, Taipei Veterans General Hospital, Taiwan

## Abstract

A combination therapy of pentoxifylline with an angiotensin converting enzyme inhibitor (ACEI) or an angiotensin II receptor blocker (ARB) decreased proteinuria or glomerular filtration rate decline in early chronic kidney disease (CKD). Whether adding pentoxifylline to ACEI/ARB provides additional benefits on outcome is unclear in CKD stage 5 patients who have not yet received dialysis (CKD 5 ND). A prospective cohort study was conducted based on the Taiwan National Health Insurance Research Database. From January 1, 2000 to June 30, 2009, we enrolled 14,117 CKD 5 ND with serum creatinine levels >6 mg/dL and hematocrit levels <28% and who have been treated with ACEI/ARB. All patients were divided into pentoxifylline users and nonusers. Patient follow-up took place until dialysis, death before initiation of dialysis or December 31, 2009. Finally, 9,867 patients (69.9%) required long-term dialysis and 2,805 (19.9%) died before dialysis. After propensity score-matching, use of pentoxifylline was associated with a lower risk for long-term dialysis or death in ACEI/ARB users (HR, 0.94; 95% CI, 0.90–0.99) or ARB users (HR, 0.91; 95% CI, 0.85–0.97). In conclusion, pentoxifylline exhibited a protective effect in reducing the risk for the composite outcome of long-term dialysis or death in ACEI/ARB treated CKD 5 ND.

The increasing global prevalence of chronic kidney disease (CKD) has profound impacts on public health and economic problems^1^. Activation of the renin-angiotensin-aldosterone system (RAAS) and the production of growth factors and inflammatory mediators such as platelet-derived growth factor, transforming growth factor ß1 (TGF-ß1), tumor necrosis factor α (TNF-α), and monocyte chemoattractant protein 1 (MCP-1) play pivotal roles in CKD progression^2^,^3^,^4^,^5^. Cumulative evidence strongly recommends RAAS blockade, primarily using an angiotensin converting enzyme inhibitor (ACEI) or an angiotensin II receptor blocker (ARB), as the first-line antihypertensive agents for the treatment of CKD^6^. Inhibition of the RAAS with an ACEI or an ARB not only delays the progression of CKD both in diabetic and non-diabetic stage 1–3 CKD patients^7^,^8^,^9^,^10^, but also in non-diabetic stage 4 CKD in randomized control trials^11^,^12^. Recently, our study also disclosed that the use of RAAS blockade in pre-dialysis stage 5 CKD was associated with lower risk for long-term dialysis^13^. However, most patients eventually progress to end-stage renal disease (ESRD) even after intensive use of RAAS blockade. Therefore, it is important to find new strategies, such as combining RAAS blockade with another agent, targeting inflammatory pathways to arrest CKD progression.

Pentoxifylline, a nonselective phosphodiesterase inhibitor, exerts potent inhibitory effects against cell proliferation, inflammation, and extracellular matrix accumulation^14^,^15^,^16^. Monotherapy with pentoxifylline markedly decreased proteinuria and was associated with a decrease in urinary TNF-α and MCP-1 excretion in patients with proteinuric diabetic and non-diabetic kidney disease^17^,^18^. Few randomized, controlled studies proved its add-on efficacy in decreasing proteinuria or glomerular filtration rate (GFR) decline in stage 3 and 4 nondiabetic or diabetic CKD patients using RAAS blockade^19^,^20^,^21^. However, whether adding pentoxifylline to ACEI/ARB provides additional benefits on renal outcome or survival remains unclear in CKD stage 5 patients who have not yet received dialysis (CKD 5 ND).

According to the National Health Insurance (NHI) reimbursement regulations, CKD patients in Taiwan who had a serum creatinine level >6 mg/dL (approximately equivalent to an estimated GFR <15 ml/min per 1.73 m^2^) and a hematocrit <28% could receive ESA treatment to maintain a hematocrit level not exceeding 36%. Administering ESA provides a unique opportunity to identify a study population with CKD 5 ND who were anemic and hypertensive with an ACEI/ARB prescription. To bridge the evidence gap in the transition from stage 1 to 4 CKD to CKD 5 ND, we investigated the association between the pentoxifylline use and the risks of long-term dialysis and/or death in a nationwide population-based, propensity score-matched cohort of CKD 5 ND patients treated with ESAs and ACEI/ARB.

## Results

### Patient Characteristics

**Figure 1:**
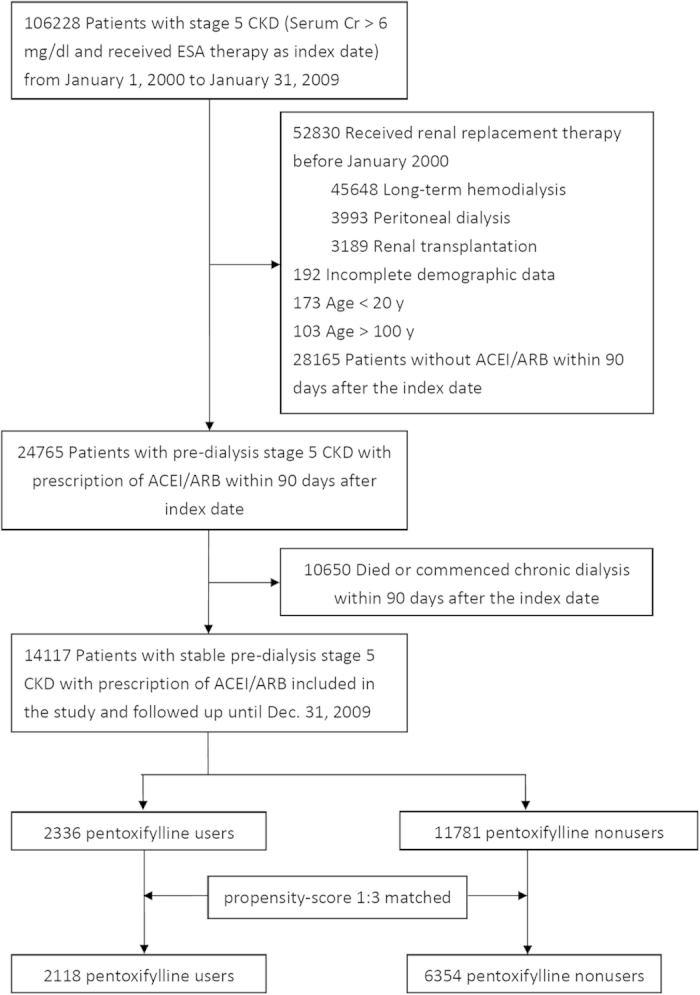
Flowchart of patient selection. Abbreviations: ACEI, angiotensin converting enzyme inhibitor; ARB, angiotensin II receptor blocker; CKD, chronic kidney disease; Cr, creatinine; ESA, erythropoiesis stimulating agent.

Figure 1 shows the flow chart for patient selection. The date of starting ESA therapy for each patient was defined as the index date. Next, 14,117 individuals with CKD 5 ND under RAAS blockade were enrolled for analysis. The detailed RAAS blockade classifications are shown in Supplemental Table 1. All patients were classified as pentoxifylline users or nonusers within 90 days after the index date. Among this population, 2,336 (16.5%) patients were pentoxifylline users and 11,781 (83.5%) were nonusers. The mean age of the users was 64.0 years, of whom 52.4% were male and 60.0% had diabetes mellitus (Table 1). Compared with the pentoxifylline nonusers, the pentoxifylline users were predominantly male, more diabetic and more likely to visit nephrologists in the preceding 3 years. Additionally, for each pentoxifylline user, we identified three nonusers from our selected cohort who were frequency-matched with propensity scores that were calculated from all of the baseline covariates. After 1 to 3 matching, the distributions of propensity score between the user and nonuser groups of pentoxifylline were comparable (Supplemental Fig. 1). The nearest-neighbor algorithm was applied to construct matched pairs, assuming that the proportion of 0.995 to 1.0 is perfect^22^. Finally, we matched 2,118 pentoxifylline users and 6,354 pentoxifylline nonusers (Fig. 1).

### Add-on Protective Effects of Pentoxifylline in CKD 5 ND Patients with ACEI/ARB Use

**Figure 2:**
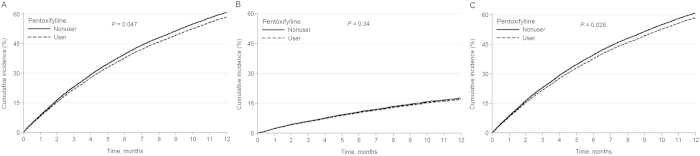
Cumulative incidence of long-term dialysis (A), death (B) and the composite outcome of long-term dialysis or death (C) among ACEI/ARB treated pre-dialysis stage 5 CKD patients. Abbreviations: ACEI, angiotensin converting enzyme inhibitor; ARB, angiotensin II receptor blocker; CKD, chronic kidney disease.

The total follow-up summation was 14,071 person-years during the study period. The mean follow up time was 11.4 months in pentoxifylline user and 12.1 months in nonuser groups. A total of 9,867 (69.9%) patients progressed to end-stage renal disease (ESRD) requiring maintenance dialysis, and 2805 (19.9%) died before progression to ESRD requiring dialysis (Table 2). The incidence of long-term dialysis was 73.0 per 100 person-years in the pentoxifylline users and 69.6 per 100 person-years in the nonusers. Adjusted survival curves revealed that patients treated with pentoxifylline were associated with a significantly lower chance for chronic dialysis (Fig. 2A). Compared with the nonusers, the pentoxifylline users were associated with a decreased risk of progression to ESRD necessitating maintenance dialysis (adjusted HR, 0.95; 95% CI 0.90–0.99) (Table 2). However, after adjusting for different covariates in the propensity score-matching model, the renal protective effect of pentoxifylline use was modest (HR, 0.94; 95% CI, 0.89–1.01) in all RAAS blockade users. We further found that add-on use of pentoxifylline to ARB monotherapy could significantly reduce the risk of chronic dialysis (HR, 0.91; 95% CI, 0.85–0.98) compared with pentoxifylline nonusers in the propensity score-matching model. However, the renal protective effects were neutral with ACEI monotherapy (HR, 0.98; 95% CI, 0.87–1.11) or concurrent ACEI and ARB use (HR, 1.03; 95% CI, 0.87–1.22).

**Figure 3:**
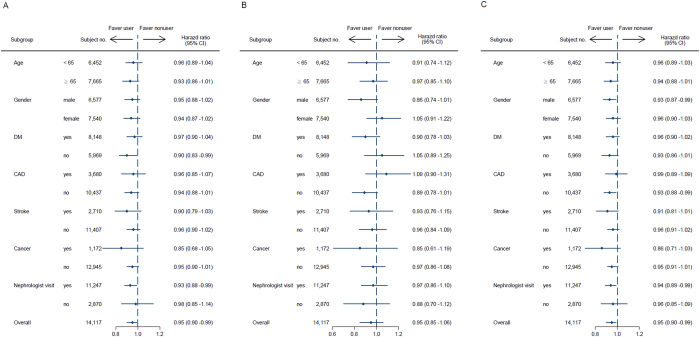
Adjusted hazard ratios of long-term dialysis (A), death (B) and the composite outcome of long-term dialysis or death (C) among ACEI/ARB treated pre-dialysis stage 5 CKD patients by pentoxifylline use. Abbreviations: ACEI, angiotensin converting enzyme inhibitor; ARB, angiotensin II receptor blocker; CAD, coronary artery disease; CI, confidence interval; CKD, chronic kidney disease; DM, diabetes mellitus; HR, hazard ratios.

The association between pentoxifylline administration and pre-dialysis death was also evaluated (Table 2). The incidence of death was 18.5 per 100 person-years in the pentoxifylline users and 20.2 per 100 person-years in the nonusers. However, adjusted survival curves revealed that patients treated with pentoxifylline were not significantly associated with death (Fig. 2B). The effect of pentoxifylline use on survival advantage was modest without statistical significance in the propensity score-matching model (HR, 0.95; 95% CI, 0.85–1.06) (Table 2). Furthermore, adjusted survival curves demonstrated that patients treated with pentoxifylline were associated with a significantly lower chance for the composite outcome of long-term dialysis or death (Fig. 2C). We found that add-on use of pentoxifylline was associated with a lower risk for the composite outcome of long-term dialysis or death in those treated with ACEI/ARB (HR, 0.94; 95% CI, 0.90–0.99) or ARB alone (HR, 0.91; 95% CI, 0.85–0.97) in the multivariate Cox regression (Table 2). Again the beneficial effects of pentoxifylline on the composite outcome of long-term dialysis or death persisted after adjusting for different covariates in the propensity score-matching model (HR, 0.94; 95% CI, 0.90–0.99). In the stratified analyses, the decreased HRs of chronic dialysis and the composite of chronic dialysis or death in the pentoxifylline cohort were generally consistent across all subgroups (Fig. 3).

### Sensitivity analyses

The results of the sensitivity analyses were consistent with the main findings. The estimated effects of pentoxifylline use on the primary outcomes were similar, regardless of whether we redefined the exposure time (Supplemental Tables 2, 3 and 4), used the other two different statistical methods including as-treated and time-dependent models (Supplemental Tables 5), or changed the observational periods (Supplemental Table 6).

## Discussion

The mechanism of the add-on effect of pentoxifylline might be explained by several animal and human studies revealing the effect of pentoxifylline on renal inflammation and fibrosis. Lin *et al.* disclosed that pentoxifylline could attenuate renal function progression in rats with remnant kidneys^15^. Furthermore, the authors disclosed that pentoxifylline attenuated renal fibrosis by blocking Smad3/4-activated transcription in a unilateral ureter obstruction model^16^. Navarro *et al.* found that pentoxifylline ameliorated the increase of the pro-inflammatory cytokines TNF-α, interleukin-1, and interleukin-6 in a diabetic nephropathy animal model^23^. In humans, the additive antiproteinuric effect of pentoxifylline has been clearly reported in type 2 diabetes patients with normal kidney function on RAAS blockade^24^. Limited randomized controlled studies had demonstrated the additive renoprotective effects of pentoxifylline in moderate CKD or diabetic patients. Lin *et al.* disclosed that pentoxifylline added to losartan therapy for one year decreased proteinuria in CKD patients with a mean eGFR of 40 mL/minute/1.73 m^2^ 19. Perkins *et al.* demonstrated that add-on pentoxifylline may slow the eGFR decrease in advanced CKD patients (mean eGFR of 20.5 mL/minute/1.73 m^2^)^20^. Recently, in the PREDIAN trial, the authors also showed that pentoxifylline addition to RAAS blockade resulted in a small decrease in eGFR and a greater reduction of residual albuminuria in diabetic stage 3–4 CKD patients^21^. However, our knowledge about the renal protective effect of a combination of pentoxifylline and RAAS blockade in stage 5 CKD is limited.

Few studies have investigated the long-term outcome of pentoxifylline use other than its effects on decreasing proteinuria or hastening the GFR decline rate in non-dialysis CKD. This is not surprising, as the study periods were short and all the previous studies were small in scale. Therefore, despite three randomized controlled studies on non-dialysis CKD, evidence of pentoxifylline use on hard outcomes (i.e., overall mortality or progression to ESRD necessitating long-term dialysis) in patients with predialysis CKD is lacking. To the best of our knowledge, our large-scale, nationwide cohort study is the first to demonstrate that in CKD 5 ND patients, add-on use of pentoxifylline was associated with 6% lower risk for the composite outcome of long-term dialysis or death in those treated with ACEI/ARB and 9% lower risk of initiation of chronic dialysis in ARB monotherapy. A small-scale, single-center, observational study^25^ had disclosed that the add-on use of pentoxifylline to RAAS blockade provided a better renal outcome than those treated with RAAS blockade alone in CKD patients with mean eGFR of 25–26 mL/minute/1.73 m^2^. The authors also disclosed that there were no significant differences between pentoxifylline users and non-users regarding the overall mortality in advanced CKD patients treated with RAAS blockade. Whether the result could be extrapolated to stage 5 ND was questionable. In contrast, our study not only extends the current knowledge in the field but also demonstrates the consistency and generalizability of the effectiveness of add-on pentoxifylline to RAAS blockade from patients with stage 1 to 4 CKD to CKD 5 ND.

From a clinical viewpoint, several issues merit discussion in this study. First, for patients with rapidly declining renal function and low GFR, including CKD 5 ND patients and rapidly progressing DM nephropathy patients, physicians usually do not prescribe ACEI or ARB, and these patients were excluded in many studies. This is the reason why research on pre-dialysis advanced CKD is limited. However, our previous study^13^ demonstrated that ACEI/ARB users exhibited an association with lower risk of long-term dialysis or death by 6%, but had a higher hyperkalemia-associated hospitalization rate. The risk of pre-dialysis mortality caused by hyperkalemia was not significantly increased^13^. Therefore, pentoxifylline represents a promising and inexpensive add-on agent for these patients. Second, less is known about the relative efficacy and safety of ACEI and ARB in ESRD. There have been few comparative effective studies between ACEI and ARB in ESRD. In ESRD patients, ARB induced a greater anti-inflammatory effect compared with ACEI^26^, and an ARB in combination with another antihypertensive medication may have a beneficial effect on cardiovascular mortality^27^. One potential explanation for the differential effects on the inflammatory response between ACEI and ARB may relate to their effect on bradykinin metabolism. ACEI not only prevents the formation of angiotensin II, it also potentiates the actions of bradykinin by inhibiting its degradation and by altering B2 receptor sensitivity^28^. Bradykinin induces fibrinolysis and stimulates inflammation^29^,^30^. Since oxidative stress is often accompanied by an increase in circulating pro-inflammatory cytokines in advanced CKD patients. It is possible that the add-on pro-inflammatory effects of bradykinin partially negate the beneficial effects of interrupting the RAAS system and reducing blood pressure in advanced CKD. Because our observational study may be associated with unadjusted residual confounding, randomized clinical trials are needed to validate this association. Third, ACEI and ARB combination therapy is more antiproteinuric in the short term than ACEI or ARB alone^31^. The Ongoing Telmisartan Alone and in Combination with Ramipril Global Endpoint Trial (ONTARGET) found that dual RAAS blockade was associated with a higher risk of serious adverse events such as acute kidney injury and hyperkalemia compared with ARB monotherapy^32^,^33^. Combination therapy did not give a significant benefit with respect to progression of renal disease or death. Along the same lines, our study also demonstrated no renal benefit from combining pentoxifylline and dual RAAS blockade therapy compared with pentoxifylline and ARB monotherapy.

Our study was notable for its large sample size, its nationally representative nature, and the fact that the selected cohort was validated by a strict NHI reimbursement regulation. By selecting those patients who survived longer than 90 days and following them from the end of this time window, we were able to control for survival bias^34^ However, some limitations in this study should be acknowledged. First, our study is observational in nature and cannot prove causality. To assure adequate statistical power (*α* = 0.05; 1-*β* = 0.8, no loss of follow-up), at least 38,584 CKD 5 ND patients should be enrolled to examine a 9% relative risk reduction. Apart from the impracticability of conducting such a large-scale, randomized controlled trial, emerging evidence^35^,^36^ suggests that well-designed observational studies can yield comparable outcomes. Second, some laboratory data such as proteinuria or renal function are not available in the present study. However, these confounders in our study could be minimized because the model robustness has been tested in a propensity score-based matched design. In addition, the consistent findings of sensitivity analyses assure the robustness of this study. Moreover, nephrologists usually prescribe pentoxifylline in obvious proteinuria and overt proteinuria, thus nephrologist visit is a predictor of worse renal outcome. The use of pentoxifylline in patients with more proteinuria will bias the result toward the null hypothesis. Third, it can be argued that acute renal failure patients with transient creatinine levels >6 mg/dL were likely included in our cohort. Therefore, we restricted our analysis to patients who persistently received ESA therapy at two or more consecutive ambulatory visits, and the result was not materially changed. Finally, the generalizability of our data is limited to the population of anemic, hypertensive CKD 5 ND patients under ESA and RAAS blockade use. The results may not be applicable to all CKD 5 ND patients.

In conclusion, our findings from this nationwide cohort expand the existing knowledge of pentoxifylline use from stage 1 to 4 CKD to CKD 5 ND patients who were treated with ACEI/ARB. Our cohort study reveals that add-on use of pentoxifylline to ACEI/ARB was significantly associated with lower risk of the composite outcome (chronic dialysis or death) in CKD 5 ND. Further studies with more sample size may be needed to illustrate its definite results.

## Methods

### Data source

The present study was based on data obtained from the NHI Research Database, which contains the healthcare data from more than 95% of the hospitals in Taiwan and 99% of the entire population of 23 million enrolled in the NHI program^37^. The comprehensive healthcare information maintained in the NHI Research Database included date of birth, gender, residency area, diagnostic codes, medication prescriptions, and medical procedures. International Classification of Diseases-9th revision (ICD-9) codes were used to define diseases. The study was approved by the Institutional Review Board at Taipei Tzu Chi Hospital, and the methods were carried out in strict accordance with the approved guidelines for researches involving human subjects of the Taiwan Ministry of Health and Welfare. Informed consent was waived due to personal information that had been de-identified in the NHI Research Database.

### Design and study participants

The study was designed as a population-based longitudinal cohort study to investigate the association between add-on use of pentoxifylline to RAAS blockade and the occurrence of death before initiating dialysis or chronic dialysis in CKD 5 ND patients. The orders of the NHI-reimbursed ESA, usually prescribed by nephrologists, are generally accurate because the Bureau of NHI regularly audited the claims. Individuals who had a primary diagnosis of CKD and received ESA treatments between January 1, 2000, and December 31, 2009 were selected. The cohort has been described in our previous study^13^,^38^. Based on an internal report of the Taiwan Department of Health, the ESA use rate was 85% in 2012 among stage 5 CKD patients who had not yet commenced renal replacement therapy. The median hematocrit level of incident dialysis patients was 24.2% (interquartile range, 20.6–27.5%) in Taiwan. Therefore, the selected cohort in this study was the most representative of CKD 5 ND patients in Taiwan^39^. The first day of ESA prescription was defined as the index date. We excluded patients younger than 20 years or older than 100 years of age, patients who received dialysis or renal transplantation before the index date, patients without ACEI/ARB within 90 days after index date, and patients who died or had commenced renal replacement therapy within 90 days after the index date. Comorbidities, including diabetes mellitus, coronary artery disease (CAD), stroke, and cancer, were defined as the diseases diagnosed within three years before the index date. The Charlson comorbidity index (CCI) was used to quantify patient comorbidity profiles^40^.

### Exposure assessment

The detailed ACEI/ARB information is shown in Supplemental Table 1. Patients who had taken pentoxifylline within 90 days after the index date were defined as pentoxifylline users (n = 2,336), and the remaining subjects were defined as pentoxifylline nonusers (n = 11,781).

### Outcomes

The observation period started 90 days after the index date until death or the commencement of chronic dialysis, whichever occurred first, or December 31, 2009. The primary outcomes were all-cause death before chronic dialysis or chronic dialysis. The onset of mortality was the date of death. The onset of renal outcome was defined as the date of ESRD development and the commencement of chronic dialysis for at least 90 days.

### Statistical analysis

The baseline characteristics were compared by the 2-sided *t* test and *x*^2^ test. In multivariable Cox’s proportional hazard models, the effects of pentoxifylline were adjusted for age, gender, CCI score, presence of diabetes mellitus, CAD, stroke and cancer, the number of visits to nephrologists within 3 years before the index date (0, 1–6, or >6 visits), geographic location (northern, middle, southern, or eastern/other islands, according to NHI registration locations), and anti-hypertensive medications. The study entry was defined as the 90th day after the index date. Patient follow-up visits took place until the time of dialysis, pre-dialysis death or December 31, 2009. The primary outcomes were pre-dialysis death or chronic dialysis. The results were expressed as HR for pentoxifylline users compared with nonusers. The proportional hazard assumption, the constant HR over time, was evaluated by comparing estimated log-log survival curves for all time-independent covariates. All assessed log-log survival plots graphically showed two parallel lines, indicating no violation of the assumption. The adjusted HRs for death or chronic dialysis associated with pentoxifylline use were analyzed among the subgroups based on the participants’ characteristics. Additionally, because pentoxifylline users and nonusers have different baseline characteristics, we performed a propensity score-matched analysis in which we calculated a propensity score for the likelihood of using pentoxifylline by multivariate logistic regression analysis, conditional on the baseline covariates listed in Table 1. All *P* values were two-sided, and the significance level was set at 0.05. The statistical analyses were performed using SAS version 9.2 (SAS Institute Inc., Cary, NC, USA) and STATA SE version 11.0 (Stata Corp, College Station, TX, USA).

### Subgroup and Sensitivity analyses

To assess effect modification, we did subgroup analyses in pre-specified strata of clinical interest, including age, gender, the presence or absence of diabetes mellitus, coronary artery disease, stroke, cancer, and the nephrologist care. To assess the reliability of our findings, we conducted a series of analyses defining pentoxifylline use at intervals of 30, 60 days and 120 days after the first ESA prescription to minimize misclassification bias (Supplemental Tables 2, 3 and 4). In addition, two different statistical methods including as-treated and time-dependent model were performed to assure robustness of results found in our study (Supplemental Table 5). To look for any evidence of a cohort effect due to different patients’ recruitment time, we also analyses for patients with index date from 2000–2007 (Supplemental Table 6).

## Additional Information

**How to cite this article**: Kuo, K.-L. *et al.* Add-on Protective Effect of Pentoxifylline in Advanced Chronic Kidney Disease Treated with Renin-Angiotensin-Aldosterone System Blockade - A Nationwide Database Analysis. *Sci. Rep.* **5**, 17150; doi: 10.1038/srep17150 (2015).

## Supplementary Material

Supplementary Information

## Figures and Tables

**Table 1:** Baseline characteristics of study subjects before and after propensity score matching, by pentoxifylline use^a^.

	Before matching				Propensity score-matched			
	Pentoxifylline users (n = 2336)	Pentoxifylline nonusers (n = 11781)	*P*	*SD*	Pentoxifylline users (n = 2118)	Pentoxifylline nonusers (n = 6354)	*P*	*SD*
Age, mean (SD), y	64.0 (13.1)	64.8 (13.1)	0.38	0.062	64.5 (13.0)	64.2 (13.1)	0.29	0.027
Age, group, y			0.006				0.09	
20–44	171 (7.3)	861 (7.3)		0.001	145 (6.9)	510 (8)		0.045
45–64	970 (41.5)	4,450 (37.8)		0.077	848 (40)	2,488 (39.2)		0.018
65–74	641 (27.4)	3,510 (29.8)		0.052	596 (28.1)	1,881 (29.6)		0.032
75–100	554 (23.7)	2,960 (25.1)		0.033	529 (25)	1,475 (23.2)		0.041
Gender								
Male	1,225 (52.4)	5,352 (45.4)	<0.001	0.141	1,037 (49)	3,198 (50.3)	0.28	0.027
Comorbid conditions within 3 y before the index date								
Diabetes	1,401 (60)	6,747 (57.3)	0.02	0.055	1,247 (58.9)	3,759 (59.2)	0.82	0.006
MI	613 (26.2)	3,067 (26)	0.83	0.005	558 (26.4)	1,673 (26.3)	0.99	0.001
Stroke	492 (21.1)	2,218 (18.8)	0.01	0.056	418 (19.7)	1,246 (19.6)	0.90	0.003
Cancer	170 (7.3)	1,002 (8.5)	0.049	0.046	166 (7.8)	459 (7.2)	0.35	0.023
Charlson Comorbidity Index score			0.19				0.83	
<3	794 (34)	4,216 (35.8)		0.038	740 (34.9)	2,223 (35)		0.001
4–5	857 (36.7)	4,280 (36.3)		0.007	770 (36.4)	2,347 (36.9)		0.012
>5	685 (29.3)	3,285 (27.9)		0.032	608 (28.7)	1,784 (28.1)		0.014
Mean (SD)	4.5 (2.2)	4.4 (2.3)	0.09	0.03	4.5 (2.2)	4.5 (2.2)	0.99	0.001
Nephrologist visits within 3 y before the index date			<0.001				0.81	
0	366 (15.7)	2,504 (21.3)		0.144	365 (17.2)	1,079 (17)		0.007
1–6	536 (23)	3,097 (26.3)		0.078	515 (24.3)	1,511 (23.8)		0.013
>6	1,434 (61.4)	6,180 (52.5)		0.181	1,238 (58.5)	3,764 (59.2)		0.016
Anti-hypertensive agents used								
ACEI	833 (35.7)	5,081 (43.1)	<0.001	0.153	794 (37.5)	2,387 (37.6)	0.95	0.002
ARB	1,847 (79.1)	8,460 (71.8)	<0.001	0.169	1,634 (77.2)	4,907 (77.2)	0.94	0.002
Beta-blockers	1,093 (46.8)	4,960 (42.1)	<0.001	0.094	947 (44.7)	2,902 (45.7)	0.44	0.019
Calcium channel blockers	1,731 (74.1)	8,153 (69.2)	<0.001	0.109	1,532 (72.3)	4,653 (73.2)	0.42	0.020
Diuretics	1,549 (66.3)	7,752 (65.8)	0.64	0.011	1,424 (67.2)	4,242 (66.8)	0.69	0.010
Insulin	753 (32.2)	3,322 (28.2)	<0.001	0.088	642 (30.3)	1,931 (30.4)	0.95	0.002
Statin	595 (25.5)	2,363 (20.1)	<0.001	0.129	467 (22.1)	1,449 (22.8)	0.47	0.018
Aspirin	636 (27.2)	2,606 (22.1)	<0.001	0.119	528 (24.9)	1,611 (25.4)	0.70	0.010
Acetaminophen	1,173 (50.2)	5,863 (49.8)	0.69	0.009	1,050 (49.6)	3,172 (49.9)	0.78	0.007
NSAIDs								
COX-2 inhibitors	106 (4.5)	544 (4.6)	0.87	0.004	96 (4.5)	292 (4.6)	0.90	0.003
Non-COX-2 inhibitors	858 (36.7)	4,446 (37.7)	0.36	0.021	777 (36.7)	2,351 (37)	0.79	0.007
Geographic location			<0.001				0.56	
Northern	1,146 (49.1)	4,515 (38.3)		0.218	1,089 (51.4)	3,157 (49.7)		0.035
Middle	794 (34)	2,581 (21.9)		0.272	633 (29.9)	1,968 (31)		0.024
Southern	377 (16.1)	4,360 (37)		0.486	377 (17.8)	1,176 (18.5)		0.018
Eastern or other islands	19 (0.8)	325 (2.8)		0.147	19 (0.9)	53 (0.8)		0.007
Propensity score	0.209(0.083)	0.157(0.082)	<0.001	0.636	0.194(0.012)	0.197(0.075)	0.19	0.032

Abbreviations: ACEI, angiotensin converting enzyme inhibitor; ARB, angiotensin II receptor blocker; CCI, Charlson comorbidity index; CKD, chronic kidney disease; COX-2, cyclooxygenase -2; NSAID, non-steroidal anti-inflammatory drug; SD, standardized difference. Diuretics included thiazides, loop diuretics, and potassium sparing agents.

^a^All of the data are presented as n (%) unless otherwise indicated.

**Table 2:** Risk of study outcomes by adding pentoxifylline in ACEI/ARB treatment among pre-dialysis stage 5 CKD patients.

	Pentoxifyllineuser		Pentoxifyllinenonuser		User vs. nonuser		
	n	IR	n	IR	Crude HR	Adjusted HR†	Adjusted HR*
Long-term dialysis							
ACEI/ARB	1612	73.0	8255	69.6	1.04 (0.98–1.09)	0.95 (0.90–0.99)	0.94 (0.89–1.01)
ARB monotherapy	1057	72.6	4817	72.5	0.99 (0.93–1.06)	0.91 (0.85–0.97)	0.91 (0.85–0.98)
ACEI monotherapy	345	72.1	2279	62.7	1.14 (1.02–1.28)	1.03 (0.92–1.16)	0.98 (0.87–1.11)
ACEI and ARB combination	210	76.2	1159	73.3	1.02 (0.88–1.18)	0.99 (0.85–1.15)	1.03 (0.87–1.22)
Death							
ACEI/ARB	409	18.5	2396	20.2	0.92 (0.83–1.02)	0.95 (0.85–1.06)	0.95 (0.85–1.06)
ARB monotherapy	232	15.9	1193	18	0.90 (0.78–1.03)	0.90(0.78–1.04)	0.90 (0.77–1.05)
ACEI monotherapy	97	20.3	791	21.8	0.93 (0.75–1.15)	1.06 (0.86–1.32)	1.09 (0.86–1.37)
ACEI and ARB combination	80	29	412	26.1	1.10 (0.87–1.40)	0.93 (0.73–1.19)	0.94 (0.72–1.23)
Long–term dialysis or death							
ACEI/ARB	2021	91.5	10651	89.8	1.01 (0.96–1.06)	0.95 (0.90–0.99)	0.94 (0.90–0.99)
ARB monotherapy	1289	88.6	6010	90.5	0.97 (0.92–1.04)	0.91 (0.85–0.97)	0.91 (0.85–0.97)
ACEI monotherapy	442	92.4	3070	84.4	1.09 (0.98–1.2)	1.04 (0.94–1.15)	0.99 (0.89–1.11)
ACEI and ARB combination	290	105.2	1571	99.4	1.04 (0.92–1.18)	0.98 (0.86–1.11)	1.01 (0.88–1.17)

Abbreviations: ACEI, angiotensin converting enzyme inhibitor; ARB, angiotensin II receptor blocker; CI, confidence interval; CKD, chronic kidney disease; HR, hazard ratio; IR: incidence rate, per 100 person-years.

^†^Adjusted for all variables listed in Table 1. *Adjusted for all variables listed in Table 1 after propensity-score matching.
