# Supplementary material for: Add-on Protective Effect of Pentoxifylline in Advanced Chronic Kidney Disease Treated with Renin-Angiotensin-Aldosterone System Blockade - A Nationwide Database Analysis
Source: Sci Rep. 2015 Nov 27;5:17150. doi: 10.1038/srep17150 (PMC4661470; doi:10.1038/srep17150)
Supplement: Supplementary Information [file srep17150-s1.pdf]

Add-on Protective Effect of Pentoxifylline in Advanced Chronic Kidney Disease Treated with Renin-Angiotensin-Aldosterone System Blockade - A Nationwide Database Analysis

Ko-Lin Kuo[1,2], Szu-Chun Hung[1,2], Jia-Sin Liu[3], Yu-Kang Chang[3], Chih-Cheng Hsu[3,4,5], Der-Cherng Tarng[5,6,7]

[1]Division of Nephrology, Taipei Tzu Chi Hospital, Buddhist Tzu Chi Medical Foundation; [2]School of Medicine, Tzu Chi University, Hualien; [3]Institute of Population Health Sciences, National Health Research Institutes, Taiwan; [4]Department of Health Services Administration, China Medical University, Taichung, Taiwan; [5]Institute of Clinical Medicine and [6]Department and Institute of Physiology, National Yang-Ming University, Taipei, Taiwan; and [7]Division of Nephrology, Department of Medicine, Taipei Veterans General Hospital

Supplementary Online Content

Supplemental Figure 1. Histograms of propensity score (A) before and (B) after matching.

Supplemental Table 1. The renin-angiotensin-aldosterone system blockade analyzed in the study.

Supplemental Table 2. Risks of study outcomes in ACEI/ARB treated pre-dialysis stage 5 CKD patients with pentoxifylline exposure within 30 days and an observation period beginning 30 days after the index date.

Supplemental Table 3. Risks of study outcomes in ACEI/ARB treated pre-dialysis stage 5 CKD patients with pentoxifylline exposure within 60 days and an observation period beginning 60 days after the index date.

Supplemental Table 4. Risks of study outcomes in ACEI/ARB treated pre-dialysis stage 5 CKD patients with pentoxifylline exposure within 120 days and an observation period beginning 120 days after the index date.

Supplemental Table 5. Risk of study outcomes in different statistical models by adding pentoxifylline in ACEI/ARB treatment among pre-dialysis stage 5 CKD patients.

Supplemental Table 6. Risk of study outcomes by adding pentoxifylline in ACEI/ARB treatment among pre-dialysis stage 5 CKD patients, years 2000-2007.

Supplemental Figure 1. Histograms of propensity score (A) before and (B) after matching.

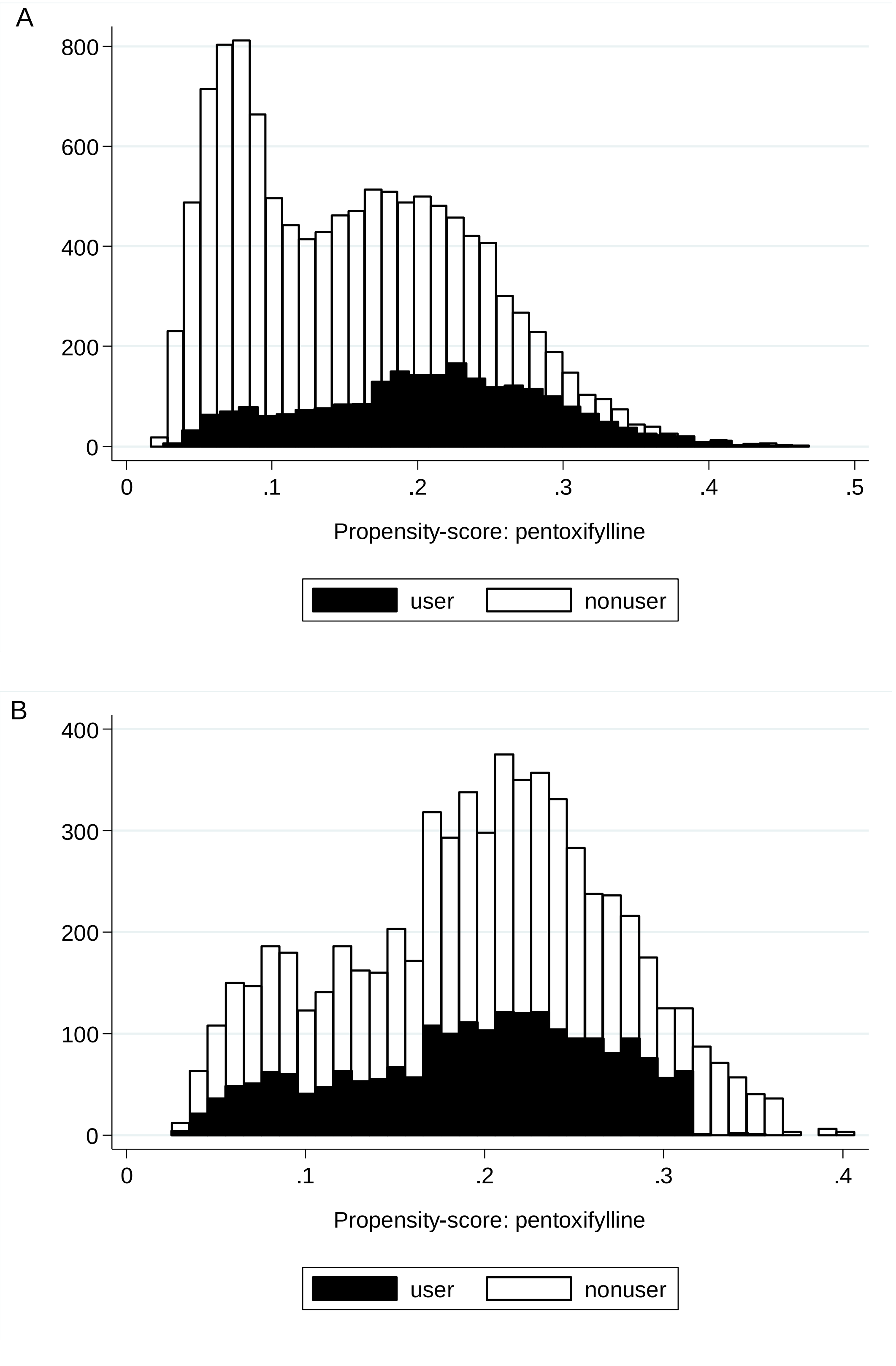

Supplemental Table 1. The renin-angiotensin-aldosterone system blockade analyzed in the study.

**Drug classification**	**Component name**
Angiotensin-converting enzyme inhibitors	Benazepril, Captopril, Cilazapril, Enalapril, Fosinopril, Imidapril, Lisinopril, Perindopril, Quinapril, Ramipril
Angiotensin II receptor blockers	Candesartan, Eprosartan, Irbesartan, Losartan, Olmesartan, Telmisartan, Valsartan

Supplemental Table 2. Risks of study outcomes in ACEI/ARB treated pre-dialysis stage 5 CKD patients with pentoxifylline exposure within 30 days and an observation period beginning 30 days after the index date.

	Pentoxifylline user N = 3216		Pentoxifylline nonuser N = 15996		User vs. nonuser	
	n	IR	n	IR	Crude HR	Adjusted HR†
**Long-term dialysis**						
ACEI/ARB	1612	62.0	12330	81.8	0.74 (0.70-0.78)	0.95 (0.89-0.99)
ARB monotherapy	1057	62.0	6846	81.8	0.74 (0.69-0.79)	0.91 (0.85-0.97)
ACEI monotherapy	345	61.6	3399	74.8	0.80 (0.72-0.89)	1.03 (0.91-1.15)
ACEI and ARB combination	210	63.1	2085	96.7	0.63 (0.55-0.73)	0.98 (0.84-1.15)
**Death**						
ACEI/ARB	409	15.7	3211	21.3	0.72 (0.65-0.80)	0.95 (0.85-1.06)
ARB monotherapy	232	13.6	1535	18.3	0.72 (0.63-0.83)	0.90 (0.78-1.04)
ACEI monotherapy	97	17.3	1064	23.4	0.72 (0.58-0.88)	1.05 (0.85-1.31)
ACEI and ARB combination	80	24.0	612	28.4	0.80 (0.63-1.01)	0.93 (0.73-1.20)
**Long-term dialysis or death**						
ACEI/ARB	2021	77.8	15541	103.2	0.73 (0.70-0.77)	0.95 (0.90-0.99)
ARB monotherapy	1289	75.5	8381	100.2	0.74 (0.69-0.78)	0.91 (0.85-0.97)
ACEI monotherapy	442	79.0	4463	98.2	0.78 (0.71-0.86)	1.03 (0.93-1.14)
ACEI and ARB combination	290	87.1	2697	125	0.67 (0.59-0.76)	0.98 (0.86-1.11)

Abbreviations: ACEI, angiotensin converting enzyme inhibitor; ARB, angiotensin II receptor blocker; CI, confidence interval; CKD, chronic kidney disease; HR, hazard ratio; IR: incidence rate, per 100 person-years.

†Adjusted for all variables listed in Table 1.

Supplemental Table 3. Risks of study outcomes in ACEI/ARB treated pre-dialysis stage 5 CKD patients with pentoxifylline exposure within 60 days and an observation period beginning 60 days after the index date.

	Pentoxifylline user N = 2832		Pentoxifylline nonuser N = 13640		User vs. nonuser	
	N	IR	n	IR	Crude HR	Adjusted HR†
**Long-term dialysis**						
ACEI/ARB	1612	67.1	10100	75.8	0.86 (0.82-0.91)	0.95 (0.89-0.99)
ARB monotherapy	1057	66.9	5753	77.4	0.85 (0.79-0.90)	0.91 (0.85-0.97)
ACEI monotherapy	345	66.5	2798	69	0.94 (0.84-1.05)	1.03 (0.91-1.15)
ACEI and ARB combination	210	69	1549	84.5	0.79 (0.68-0.91)	0.98 (0.84-1.15)
**Death**						
ACEI/ARB	409	17	2801	21	0.80 (0.72-0.89)	0.95 (0.85-1.06)
ARB monotherapy	232	14.7	1344	18.1	0.80 (0.70-0.93)	0.90 (0.78-1.04)
ACEI monotherapy	97	18.7	931	23	0.80 (0.65-0.99)	1.05 (0.85-1.31)
ACEI and ARB combination	80	26.3	526	28.7	0.89 (0.70-1.12)	0.93 (0.73-1.2)
**Long-term dialysis or death**						
ACEI/ARB	2021	84.1	12901	96.8	0.85 (0.81-0.89)	0.95 (0.90-0.99)
ARB monotherapy	1289	81.5	7097	95.5	0.84 (0.79-0.89)	0.91 (0.85-0.97)
ACEI monotherapy	442	85.2	3729	92	0.90 (0.82-0.99)	1.03 (0.93-1.14)
ACEI and ARB combination	290	95.3	2075	113.2	0.81 (0.72-0.92)	0.98 (0.86-1.11)

Abbreviations: ACEI, angiotensin converting enzyme inhibitor; ARB, angiotensin II receptor blocker; CI, confidence interval; CKD, chronic kidney disease; HR, hazard ratio; IR: incidence rate, per 100 person-years.

†Adjusted for all variables listed in Table 1.

Supplemental Table 4. Risks of study outcomes in ACEI/ARB treated pre-dialysis stage 5 CKD patients with pentoxifylline exposure within 120 days and an observation period beginning 120 days after the index date.

	Pentoxifylline user N = 2135		Pentoxifylline nonuser N = 10172		User vs. nonuser	
	n	IR	n	IR	Crude HR	Adjusted HR†
**Long-term dialysis**						
ACEI/ARB	1403	70.8	7175	67.0	1.05 (0.99-1.11)	0.95 (0.90-1.01)
ARB monotherapy	931	71.0	4231	70.6	0.99 (0.93-1.07)	0.91 (0.84-0.98)
ACEI monotherapy	296	68.9	1996	60.3	1.13 (1.01-1.28)	1.02 (0.90-1.16)
ACEI and ARB combination	176	73	948	67.8	1.07 (0.91-1.25)	1.04 (0.87-1.23)
**Death**						
ACEI/ARB	357	18.0	2048	19.1	0.95 (0.85-1.06)	0.97 (0.87-1.10)
ARB monotherapy	204	15.6	1059	17.7	0.89 (0.77-1.03)	0.89 (0.76-1.04)
ACEI monotherapy	86	20.0	652	19.7	1.02 (0.82-1.28)	1.17 (0.93-1.48)
ACEI and ARB combination	67	27.8	337	24.1	1.15 (0.89-1.50)	0.99 (0.75-1.31)
**Long-term dialysis or death**						
ACEI/ARB	1760	88.8	9223	86.2	1.03 (0.97-1.08)	0.96 (0.91-1.01)
ARB monotherapy	1135	86.6	5290	88.3	0.98 (0.92-1.04)	0.91 (0.85-0.97)
ACEI monotherapy	382	88.9	2648	80.0	1.11 (0.99-1.23)	1.05 (0.94-1.18)
ACEI and ARB combination	243	100.8	1285	91.9	1.09 (0.95-1.25)	1.03 (0.89-1.19)

Abbreviations: ACEI, angiotensin converting enzyme inhibitor; ARB, angiotensin II receptor blocker; CI, confidence interval; CKD, chronic kidney disease; HR, hazard ratio; IR: incidence rate, per 100 person-years.

†Adjusted for all variables listed in Table 1.

Supplemental Table 5. Risk of study outcomes in different statistical models by adding pentoxifylline in ACEI/ARB treatment among pre-dialysis stage 5 CKD patients.

	Intention-to-treat model	As-treated model	Time-dependent model
	Adjusted HR†	Adjusted HR†	Adjusted HR†
**Long-term dialysis**			
ACEI/ARB	0.94 (0.89-0.99)	0.93 (0.87-0.99)	0.85 (0.79-0.92)
ARB monotherapy	0.90 (0.84-0.97)	0.89 (0.82-0.96)	0.81 (0.73-0.90)
ACEI monotherapy	1.02 (0.91-1.15)	1.01 (0.88-1.16)	0.93 (0.79-1.09)
ACEI and ARB combination	0.98 (0.84-1.15)	0.99 (0.83-1.19)	0.92 (0.74-1.15)
**Death**			
ACEI/ARB	0.95 (0.85-1.06)	0.76 (0.66-0.87)	1.21 (0.98-1.50)
ARB monotherapy	0.90 (0.78-1.05)	0.80 (0.67-0.95)	1.12 (0.84-1.48)
ACEI monotherapy	1.05 (0.84-1.30)	0.59 (0.42-0.83)	1.41 (0.92-2.17)
ACEI and ARB combination	0.94 (0.73-1.21)	0.80 (0.58-1.10)	1.08 (0.63-1.87)
**Long-term dialysis or death**			
ACEI/ARB	0.94 (0.90-0.99)	0.89 (0.84-0.95)	0.94 (0.87-1.01)
ARB monotherapy	0.90 (0.85-0.96)	0.87 (0.81-0.94)	0.87 (0.79-0.95)
ACEI monotherapy	1.03 (0.93-1.14)	0.92 (0.81-1.04)	1.07 (0.92-1.24)
ACEI and ARB combination	0.98 (0.86-1.12)	0.94 (0.81-1.10)	1.01 (0.82-1.22)

Abbreviations: ACEI, angiotensin converting enzyme inhibitor; ARB, angiotensin II receptor blocker; CI, confidence interval; CKD, chronic kidney disease; HR, hazard ratio; IR: incidence rate, per 100 person-years.

†Adjusted for all variables listed in Table 1.

Supplemental Table 6. Risk of study outcomes by adding pentoxifylline in ACEI/ARB treatment among pre-dialysis stage 5 CKD patients, years 2000-2007.

	Pentoxifylline user		Pentoxifylline nonuser		User vs. nonuser		
	N	IR	n	IR	Crude HR	Adjusted HR†	Adjusted HR*
**Long-term dialysis**							
ACEI/ARB	1548	73.3	8090	70.2	1.03 (0.98-1.09)	0.94 (0.89-0.99)	0.95 (0.89-1.01)
ARB monotherapy	1015	73.1	4711	73.3	0.99 (0.92-1.06)	0.90 (0.84-0.97)	0.92 (0.85-0.99)
ACEI monotherapy	336	72.0	2246	63.1	1.13 (1.01-1.27)	1.02 (0.91-1.15)	0.98 (0.86-1.11)
ACEI and ARB combination	197	76.7	1133	74.0	1.02 (0.87-1.18)	0.98 (0.84-1.15)	1.01 (0.85-1.20)
**Death**							
ACEI/ARB	409	19.4	2396	20.8	0.93 (0.84-1.04)	0.95 (0.85-1.06)	0.98 (0.87-1.10)
ARB monotherapy	232	16.7	1193	18.6	0.91 (0.79-1.04)	0.90 (0.78-1.05)	0.95 (0.82-1.11)
ACEI monotherapy	97	20.8	791	22.2	0.93 (0.75-1.15)	1.05 (0.84-1.30)	1.04 (0.82-1.31)
ACEI and ARB combination	80	31.1	412	26.9	1.14 (0.90-1.45)	0.94 (0.73-1.21)	0.92 (0.70-1.21)
**Long-term dialysis or death**							
ACEI/ARB	1957	92.7	10486	91.0	1.01 (0.96-1.06)	0.94 (0.90-0.99)	0.95 (0.90-1.01)
ARB monotherapy	1247	89.8	5904	91.8	0.97 (0.92-1.03)	0.90 (0.85-0.96)	0.92 (0.86-0.99)
ACEI monotherapy	433	92.8	3037	85.3	1.08 (0.97-1.19)	1.03 (0.93-1.14)	0.99 (0.89-1.11)
ACEI and ARB combination	277	107.8	1545	100.9	1.05 (0.92-1.19)	0.98 (0.86-1.12)	0.99 (0.86-1.15)

Abbreviations: ACEI, angiotensin converting enzyme inhibitor; ARB, angiotensin II receptor blocker; CI, confidence interval; CKD, chronic kidney disease; HR, hazard ratio; IR: incidence rate, per 100 person-years.

†Adjusted for all variables listed in Table 1. *Adjusted for all variables listed in Table 1 after propensity-score matching.
